# X-ray-Induced Pyroelectric Effect in a Perovskite Ferroelectric Drives Low Detection Limit Self-Powered Responses

**DOI:** 10.1021/acscentsci.3c01274

**Published:** 2023-12-11

**Authors:** Yu Ma, Wenjing Li, Yi Liu, Wuqian Guo, Haojie Xu, Shiguo Han, Liwei Tang, Qingshun Fan, Junhua Luo, Zhihua Sun

**Affiliations:** † State Key Laboratory of Structural Chemistry, Fujian Institute of Research on the Structure of Matter, Chinese Academy of Sciences, Fuzhou, Fujian 350002, People’s Republic of China; § University of Chinese Academy of Sciences, Chinese Academy of Sciences, Beijing 100039, People’s Republic of China

## Abstract

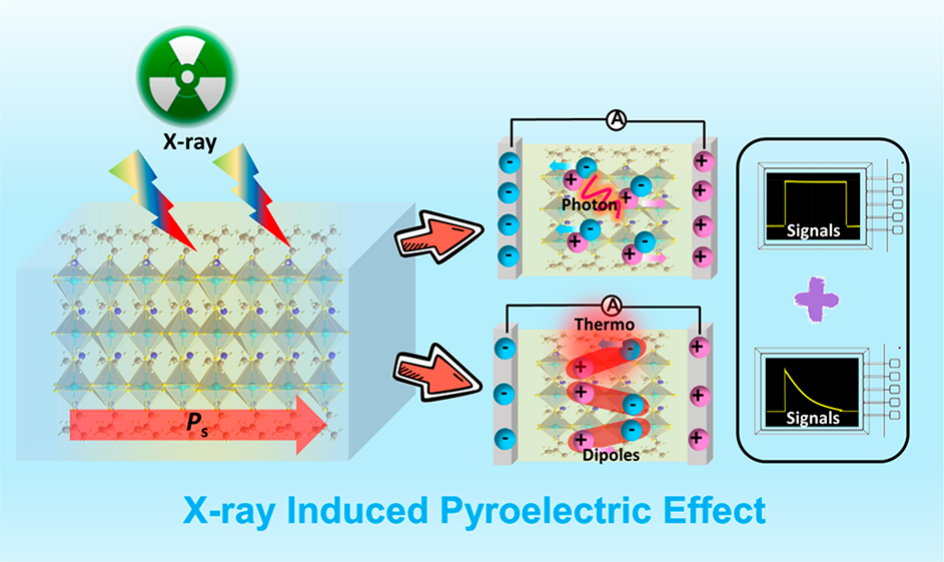

The light-induced pyroelectric effect (LPE) has shown a great promise in the application of optoelectronic devices, especially for self-powered detection and imaging. However, it is quite challenging and scarce to achieve LPE in the X-ray region. For the first time, we report X-ray LPE in a single-phase ferroelectric of (NPA)_2_(EA)_2_Pb_3_Br_10_ (**1**, NPA = neopentylamine, EA = ethylamine), adopting a two-dimensional trilayered perovskite motif, which has a large spontaneous polarization of ∼3.7 μC/cm^2^. Its ferroelectricity allows for significant LPE in the wavelength range of ordinary visible light. Strikingly, the X-ray LPE is observed in **1**, which endows remarkable self-powered X-ray responses at 0 bias, including sensitivity up to 225 μC Gy^–1^ cm^–2^ and a low detection limit of ∼83.4 nGy s^–1^, being almost 66 times lower than the requirement for medical diagnostics (∼5.5 μGy s^–1^). This work not only develops a new mode for X-ray detection but also provides valuable insights for future photoelectric device application.

## Abstract 2

For the first time, this work presents the X-ray LPE in a single-phase ferroelectric, which develops a new mode for X-ray detection and provides valuable insights for photoelectric device application.

## Introduction

X-ray detectors play an essential role in fields such as medical imaging, nondestructive testing, and space exploration due to the high energy and powerful penetrating capabilities of X-ray photons.^1−3^ Over the past few decades, conventional semiconductor materials such as silicon (Si), amorphous selenium (α-Se), and cadmium zinc telluride (CdZnTe) have been utilized in X-ray detection applications.^4−6^ However, these materials remain with limited carrier collection efficiency and sensitivity, often requiring operation under high electric fields leading to significant ion migration.^7,8^ Consequently, the development of self-powered devices that can operate without external bias voltage has attracted interest in X-ray detection. Traditionally, the construction of *p-n* heterojunctions enables the formation of built-in electric fields for the separation and transmission carriers produced by photons, thus rendering them self-powered. This approach is constrained by complex manufacturing processes and interface engineering.^9−11^ In contrast, ferroelectric materials offer a promising alternative, capable of generating controllable high voltage through the ferroelectric photovoltaic effect within a single-phase material for triggering optical detection.^12−14^ Notably, the spontaneous polarization in ferroelectric materials can be configured and modified through electric field and temperature, thereby enabling a wide range of electrically tunable and controllable functionalities.^15−17^

The light-induced pyroelectric effect (LPE), which combines photovoltaic and photoexcited pyroelectricity, has proven to be an efficient method of controlling the charge carrier behavior of optoelectronic devices.^18−20^ This one-of-a-kind light–matter interaction enables both stable photovoltaic current and instantaneous pyroelectric current; the former is generated by polarization electric field, whereas the latter is caused by redistribution of thermally induced pyroelectric charges in the polar direction.^21,22^ Two-dimensional hybrid perovskite ferroelectrics benefit from the coupling between spontaneous polarization and unique physical properties, including large absorption coefficients and excellent charge transport, making them suitable candidates for LPE research, such as (*n*-hexylammonium)_2_CsPb_2_Br_7_, (isoamylammonium)_2_(EA)_2_Pb_3_Cl_10_, and (*p*-bromobenzylammonium)_2_(EA)_2_Pb_3_Br_10_.^23−25^ Structurally, it consists of interleaved inorganic and organic interlayers, which not only provide pathways for carrier transport within the traps but also offer organic barriers to suppress ion migration, thereby minimizing dark current and enhancing stability.^26,27^ Notably, the diverse organic components surrounded by the inorganic layers allow greater freedom of movement, leading to the ordered arrangement of molecular dipoles, which facilitates the generation of ferroelectricity and enables self-powering.^28,29^ While research on these two-dimensional ferroelectric materials as optically active candidates is steadily progressing, LPE investigations have so far been limited to the visible-infrared region and have not been explored in the X-ray region. In this context, achieving LPE in the X-ray region holds significant promise for the development of an innovative self-powered X-ray detection mode.

In this work, for the first time, X-ray LPE is explored in a single-phase perovskite ferroelectric (NPA)_2_(EA)_2_Pb_3_Br_10_ (**1**, NPA = neopentylamine, EA = ethylamine), which has a large spontaneous polarization ∼3.7 μC/cm^2^. Its ferroelectricity enables significant LPE in the wavelength range of ordinary visible light. Surprisingly, X-ray LPE is discovered in **1**, which exhibits strong self-powered X-ray responses at 0 bias, including sensitivity up to 225 μC Gy^–1^ cm^–2^ and a low detection limit of 83.4 nGy s^–1^. And X-ray LPE can generate a sharp current peak ∼16 nA/cm^2^, which is nearly 2 orders of magnitude better than some heterojunction-based self-powered devices. These findings provide new insights into the design of novel self-powered X-ray optoelectronic devices.

## Results and Discussion

A bulk crystal of **1** with a size up to 3 × 2.0 × 1.5 mm^3^ was grown from a saturated hydrobromic acid solution with a stoichiometric ratio (Figure S1) using a solution cooling method. According to single-crystal X-ray diffraction investigation, it features a typical Ruddlesden–Popper trilayered architecture with ordered organic NPA^+^ cations positioned between the inorganic layers and EA^+^ cations residing in octahedral cages. Notably, the distorted arrangement of the PbBr_6_ octahedra of the {Pb_3_Br_10_} inorganic layers implements the symmetry breaking, which is essential for the subsequent self-powered X-ray detection. Structurally, the transition from ordered to disordered of organic NPA^+^ and EA^+^ cations, as well as tilting of the inorganic PbBr_6_ octahedra, provide the driving force for the phase transition. It is proposed that the synergy of the organic and inorganic components will be advantageous for the design of novel ferroelectric semiconductors.

**Figure 1:**
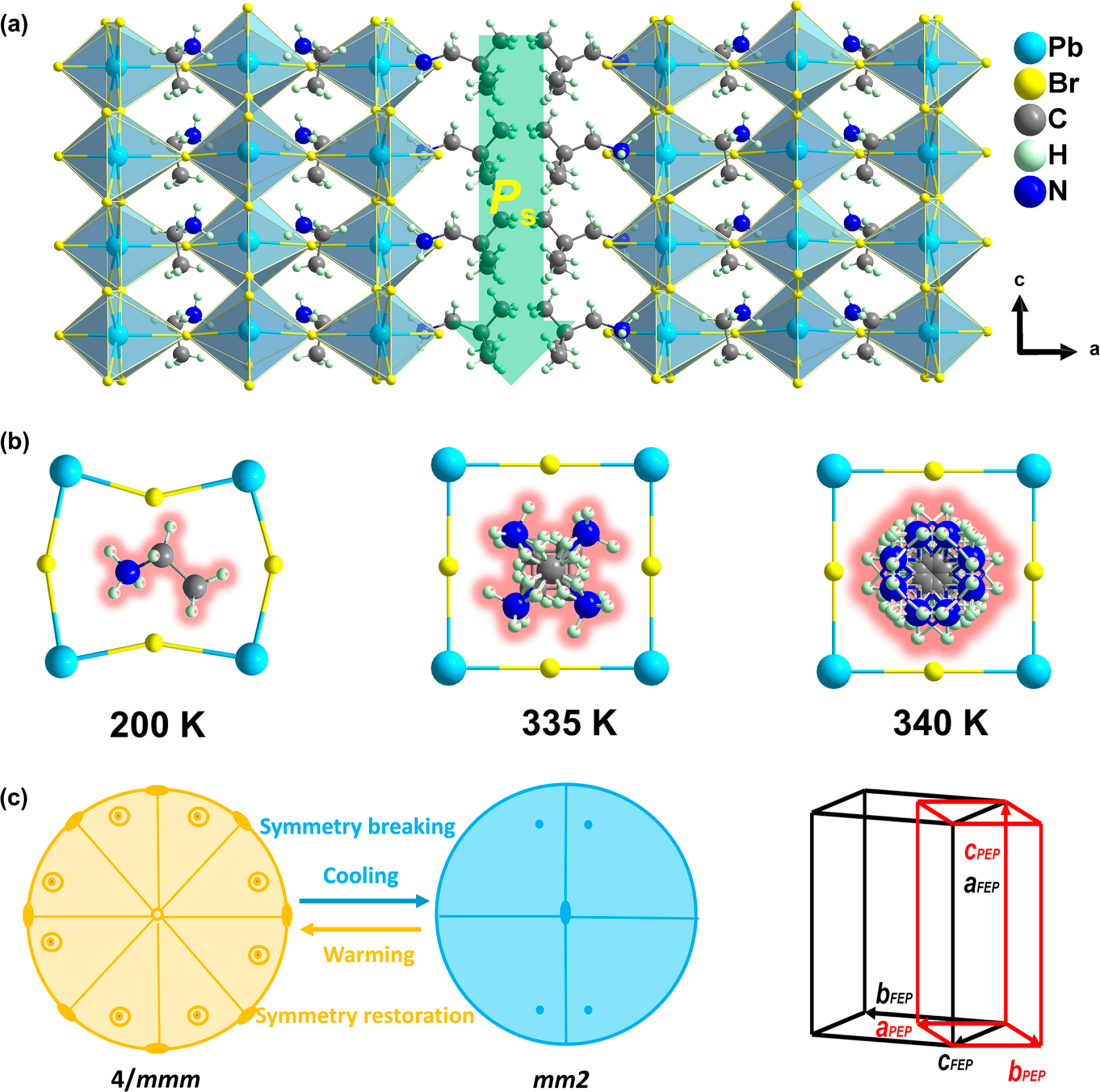
Structure of **1**. (a) Packing diagram of its crystal structure at LTP. (b) Organic EA^+^ cations are tightly confined within the perovksite cavities composed of the corner-shared octahedra at 200, 335, and 340 K. (c) Symmetry breaking for **1**. Relationship between the ferroelectric phase (FEP) unit cell (black line) and the paraelectric phase (PEP) unit cell (red line) of **1**.

**Figure 2:**
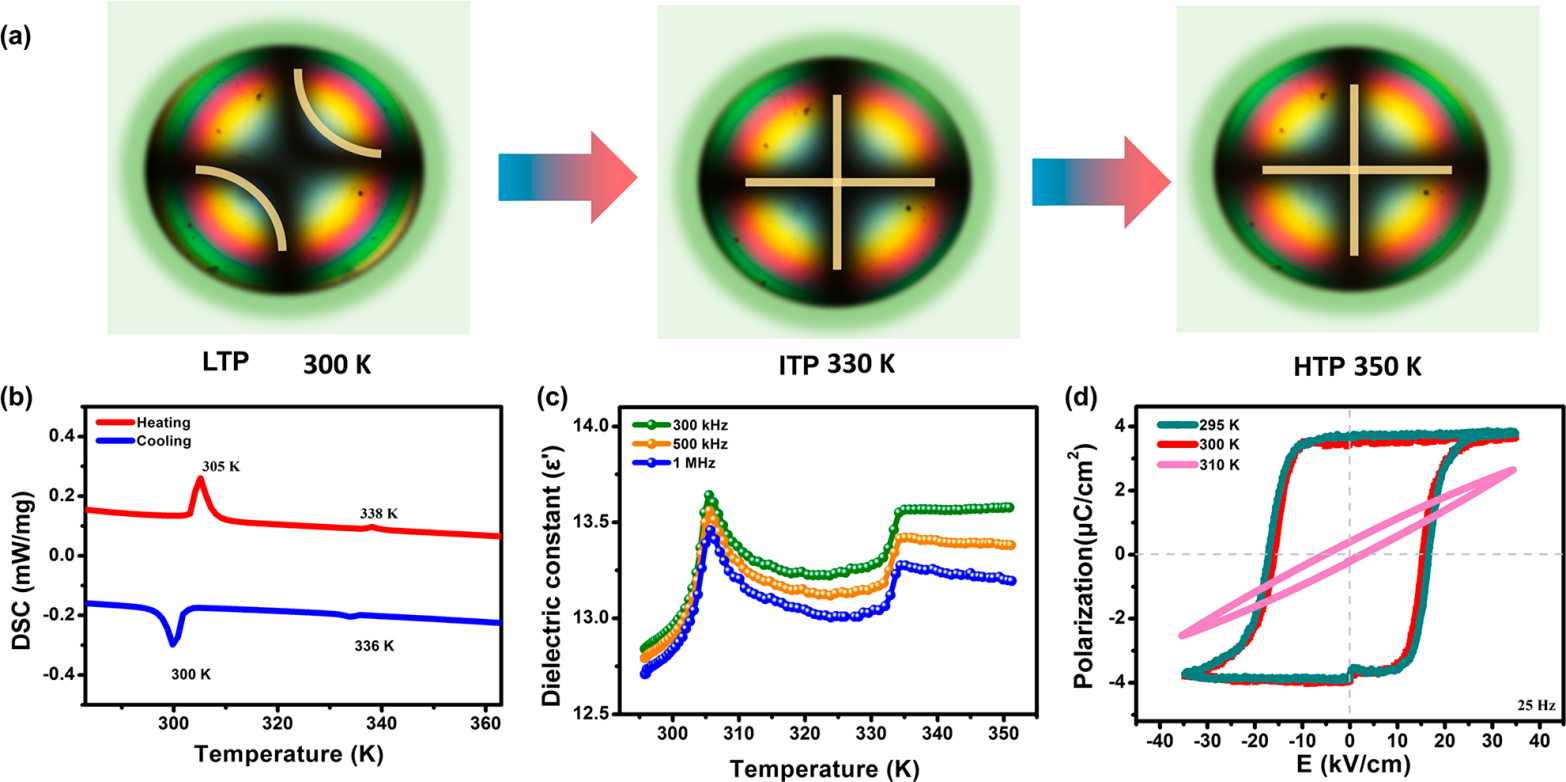
Ferroelectric properties of **1**. (a) The optical axes of **1** at different temperatures. (b) DSC curves of **1**. (c) Variable-temperature dielectric results of **1**. (d) *P*–*E* hysteresis loops measured at different temperatures.

The single-crystal X-ray diffraction was used to determine the crystal structure of **1** at different temperatures to study its phase transition behavior. In the low-temperature phase (LTP), it crystallizes in the orthorhombic system (Table S1) with the space group *Cmc*2_1_ (polar point group *mm*2). As depicted in Figure 1a, the inorganic layers are perpendicular to their ⟨100⟩ crystal face, and the bilayer organic cations of NPA^+^ are located in the spacer layer and connected to the inorganic layer through N–H···Br hydrogen bonding, with an oriented arrangement in the *c* direction. It is noteworthy that the ordered organic EA^+^ cations are completely wrapped within the cavities composed of corner-shared octahedra (Figure 1b), whereas the large-sized EA^+^ cations twist the PbBr_6_ octahedra severely, as shown through the twisted Br–Pb–Br bond angles and asymmetric Pb–Br bond lengths (Tables S2 and S3). These distortions break the inversion symmetry and promote the formation of molecular dipoles as well as the electric polarization, similar to the lead-free ferroelectric [3,3-difluorocyclobutylammonium]_2_CuCl_4_ (0.29 μC/cm^2^).^30−32^ As the temperature increases to the intermediate temperature phase (ITP), **1** crystallizes in the tetragonal system (Figure S2a) with the space group *I*4/*m* (nonpolar point group 4/*m*), characterized by disordering of the organic NPA^+^ and EA^+^ cations and symmetric configuration of the PbBr_6_ octahedra. Upon further heating to the high-temperature phase (HTP), **1** crystallizes in the tetragonal system (Figure S2b) with the space group *I*4/*mmm* (nonpolar point group 4/*mmm*). Its inorganic layer framework is similar to that of the ITP, adopting a highly symmetrical configuration, while the disorder degree of the organic cations becomes higher. All C and N atoms of the NPA^+^ and EA^+^ cations are symmetrically distributed on both sides of the mirror plane. This centrosymmetric arrangement removes the polarization of **1**, corresponding to the paraelectric phase. Therefore, the structural analysis follows the Aizu symmetry breaking of 4/*mmm*F*mm*2 during the phase transition (Figure 1c).^33^ In addition, the temperature dependence of the optical axis change reveals the characteristics of symmetry breaking. As shown in Figure 2a, two optical axes are clearly observed in the LTP, revealing the optical biaxial properties of **1**. When the temperature increases to the ITP and HTP, it displays optically uniaxial, which is consistent with the variable-temperature crystal structure analysis.

Temperature-induced symmetry breaking is an essential characteristic of ferroelectric phase transitions and a prerequisite for achieving X-ray LPE.^34^ We investigated the phase transition behavior of **1** using differential scanning calorimetry (DSC), variable-temperature dielectric constant measurements, and temperature-dependent domain patterns. As shown in Figure 2b, the DSC curves display two pairs of thermodynamic peaks, indicating **1** has two reversible phase transitions. Additionally, variable-temperature permittivity studies along the crystal polar axis (*c*-axis) verified the presence of reversible phase transitions with large dielectric anomalies at 305 and 338 K (Figure 2c). Furthermore, polarizing microscopy was used to observe cross-stripe domain patterns that vanish at temperatures over 305 K, which is consistent with the ferroelectric phase transition (Figure S3). Polarization–electric field (*P–E*) hysteresis loop measurements approved the ferroelectricity of **1** (Figure 2d and Figure S4). At room temperature, a standard *P–E* hysteresis loop with a *P*_s_ of 3.7 μC/cm^2^ can be observed along the *c*-axis, which is comparable to some recently reported ferroelectric materials.^35,36^ To our knowledge, this excellent ferroelectricity may promote the spontaneous separation of charge carriers, making self-powered detection potentially feasible.

Based on the UV–vis absorption spectrum (Figure S5), the band gap of **1** is estimated to be ∼2.74 eV, consistent with the calculation result. This is a reasonably modest bandgap for X-ray detection, which is advantageous for decreasing thermal noise and boosting the device detection performance.^37^ Meanwhile, density functional theory (DFT) calculations (Figure S6) show that **1** is a direct bandgap semiconductor, which is mainly determined by Pb s/p and Br p orbitals, i.e., the inorganic component of the hybrid perovskite contributes significantly to the bandgap. Further analysis of the charge density distribution also indicates this (Figure S7).

**Figure 3:**
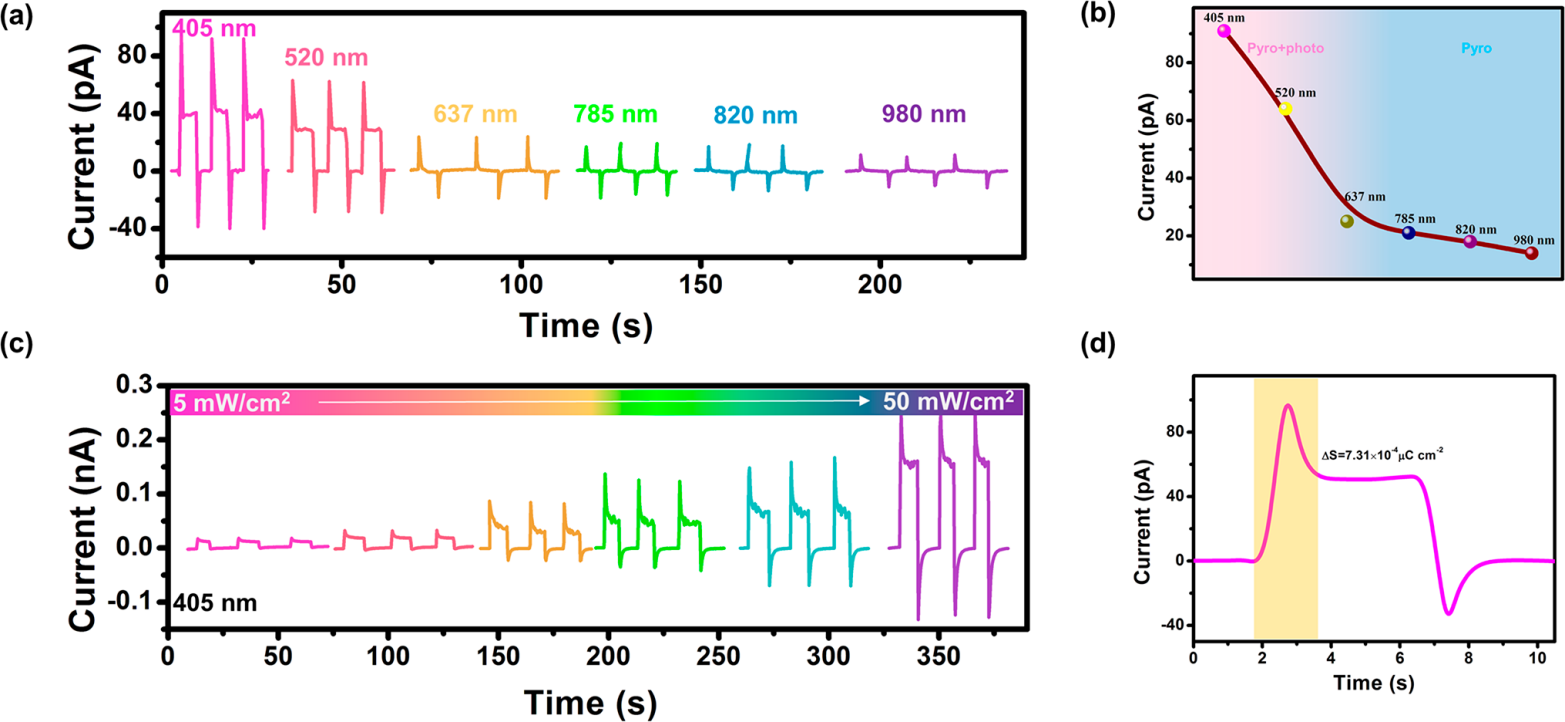
LPE of **1**. (a) Light-induced pyroelectric currents of different wavelengths (at 20 mW/cm^2^). (b) The extracted photopyroelectric peak currents as a function of incident wavelength. (c) The corresponding photopyroelectric current curves under 405 nm irradiation (5–50 mW/cm^2^). (d) One photoresponse period under 405 nm light illumination.

For ferroelectrics, spontaneous electric polarization would provide an ultrahigh built-in electrostatic field, which might accelerate the migration of photoexcited charge carriers and enable an exciting bulk photovoltaic effect.^38^ In theory, light-induced pyroelectricity is directly related to the fluctuation of ferroelectric *P*_s_, which results in the compensatory current, pyroelectric current.^39^ As a result, the coexistence of ferroelectricity and photoelectric characteristics motivates us to investigate the LPE of **1**. The apparent light-induced pyroelectric current of the crystal-based detector **1** can be clearly seen in Figure 3a during laser irradiation ranging from 405 to 980 nm. At the same radiation intensity (20 mW/cm^2^), the photocurrent exhibits wavelength-dependent behavior (Figure 3b). As an example, the photoexcited pyroelectric characteristics under 405 nm illumination are studied here. With the enhancement of laser power, the photopyroelectric current increases monotonically (Figure 3c). In addition, we investigate the thermal equilibrium process of **1** while it is illuminated. The integrated area of yellow on the heating section schematic is 7.31 × 10^–4^ μC cm^–2^ for one photoresponse period under 405 nm laser light, as illustrated in Figure 3d. The measurable temperature change in the sample using thermographic techniques is 0.9 K (Figure S8). As a result, the pyroelectric coefficient at 20 mW/cm^2^ is calculated to be 8.12 × 10^–4^ μC cm^–2^ K^–1^. These findings reveal the potential of **1** as new self-powered detectors through the LPE.

**Figure 4:**
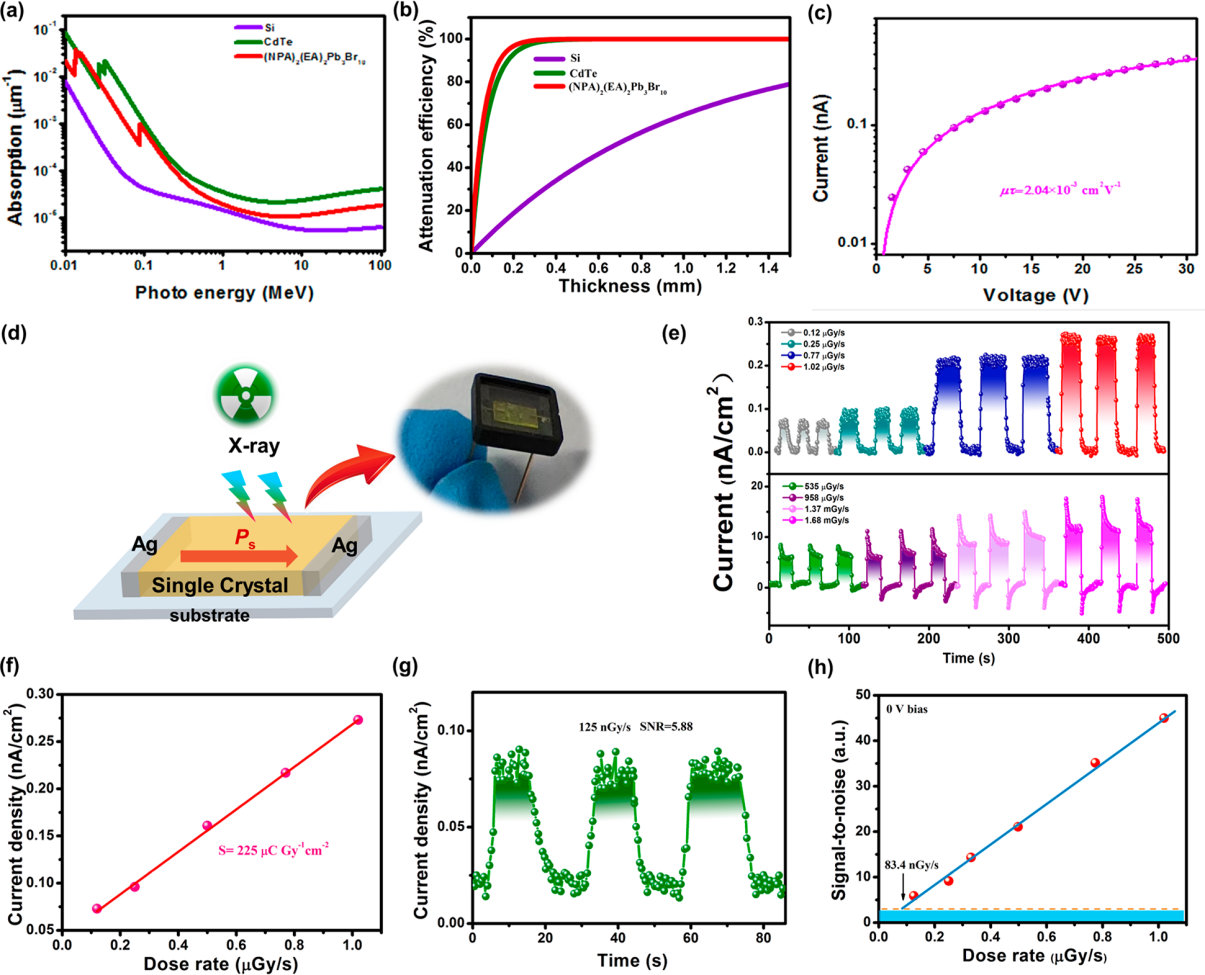
X-ray-related properties of **1**. (a) The photon energy dependence of the absorption coefficients of **1**, Si, and CdTe. (b) The X-ray attenuation efficiency versus thickness. (c) Strong X-ray photoresponse along the ferroelectric polarization direction (*c*-axis) of **1**. (d) Schematic diagram of the X-ray device based on a single crystal; the figure on the right is the physical diagram of the packaged device. (e) *I*–*t* curves under X-ray irradiation with different dose rates at 0 bias. (f) Photocurrent density at different dose rates. (g) *I*–*t* curves of device **1** under X-ray irradiation. The SNR value was also calculated. (h) X-ray dose-rate-dependent SNR at 0 V bias.

Given the exceptional performance of the LPE of **1** under normal light illumination, we expect to introduce the LPE into the X-ray region, aiming to achieve high-performance self-powered X-ray detection. The absorption spectra of **1** was investigated because the substantial absorption capacity of X-ray photons is essential for extremely sensitive X-ray detection.^40^ As depicted in Figure 4a, the X-ray absorption of **1** in the wide energy range of X-rays is far greater to that of Si and equivalent to that of CdTe. Specifically, due to the high atomic numbers of the lead and bromine atoms, the X-ray attenuation efficiency of **1** is significantly greater than that of Si (Figure 4b). This indicates that **1** has great potential for generating photogenerated carriers under X-ray irradiation. One of the most crucial factors impacting the sensitivity of X-ray detectors is carrier drift duration, which is dominated by the *μτ*.^41^ As depicted in Figure 4c, a strong X-ray photoresponse is observed along the ferroelectric polarization direction (*c*-axis) of **1**, and an extremely high *μτ* product of 2.04 × 10^–3^ cm^2^ V^–1^ is obtained. This value is substantially greater than that of standard α-Se and equivalent to that of single-crystal CH_3_NH_3_PbBr_3_.^42,43^ Moreover, Figure S9 also depicts the current density–voltage curve along the single-crystal *c*-axis of **1**, resulting in a high bulk resistivity (ρ) of 5 × 10^10^ Ω cm. This value is 100 times that of the 3D MAPbX_3_ (X = Cl, Br, or I) perovskite single crystal (10^7^–10^8^ Ω cm).^44^ This level of resistivity efficiently reduces dark current and current noise, which is necessary for steady, high-performance X-ray photodetection.

Given the exceptional ferroelectric features of single-crystal **1**, including an outstanding X-ray absorption coefficient, a large *μτ* product, and high resistivity, a self-powered X-ray detector with good performance may be expected. We fabricated and encased a passive device with a planar structure composed of Ag/**1**/Ag (Figure 4d). The detection performance was evaluated using a silver target X-ray tube and X-ray photons at energies as high as 50 keV, with a peak intensity of 22 keV. The *I–V* curves of **1** were recorded at various dosage rates in both dark and X-ray illuminated conditions. Under X-ray irradiation, a manufactured device of **1** can produce a significant open-circuit voltage ∼0.5 V (Figure S10), suggesting the presence of a bulk photovoltaic effect along the *c*-axis in the single crystal. This bulk photovoltaic effect is caused by inherent spontaneous polarization in the ferroelectric crystal structure, which enables the separation and transmission of charge carriers created by light, thereby endowing **1** with a self-powered detection capability. Indeed, under 0 V bias, the single crystal of **1** exhibits a notable X-ray response. Lower X-ray dose rates (below μGy s^–1^) resulted in a well-defined linear rise with a noticeable photocurrent plateau, confirming the excellent photoresponse to X-rays. Interestingly, when the X-ray dose rate was significantly increased, a momentary increase in peak current was observed upon opening the X-ray light source, reaching a maximum of 16 nA/cm^2^, which is 2 orders of magnitude higher than self-powered devices with heterojunction structures such as Au/CsPbBr_3_/ITO.^45^ The peak current was then gradually reduced, resulting in a steady photocurrent plateau. When the X-ray light source was turned off, a reverse current peak could be seen, followed by a slow decrease (Figure 4e). Further analysis of the measurable temperature fluctuation on the sample surface indicated that there was no change in surface temperature throughout the X-ray light switching process at low dose rates. However, under high dose rates (1.68 mGy s^–1^), a maximum temperature variation of 0.3 K was observed (Figure S11). As a result, we hypothesize that the observed peak current at high dose rates is due to an instantaneous temperature change during the light switching process, which causes a change in spontaneous polarization within the crystal, resulting in the generation of a sharp current.

Sensitivity (S) is a crucial parameter for evaluating the photonic response of X-ray detectors.^46^ A sensitivity of 225 μC Gy^–1^ cm^–2^ (Figure 4f) at 0 V bias voltage is determined by fitting the slope, which significantly surpasses the quality factor of other heterojunction-driven X-ray detectors.^47,48^ The detection limit is another critical parameter of X-ray detectors, particularly significant for practical applications such as medical diagnosis.^49^ To assess the detection limit of detector **1**, the signal-to-noise ratio (SNR) was calculated for different dose rates based on its current–time (*I–t*) curves under low-dose-rate X-ray irradiation. It is evident that at a low dose rate of 125 nGy s^–1^, the SNR for this device is 5.88 (Figure 4g). Furthermore, by further fitting the correlation between SNR and dose rate, when the dose rate is 83.4 nGy s^–1^, the SNR is calculated as 3 (Figure 4h). Thus, the detection limit for detector **1** was determined to be 83.4 nGy s^–1^, which is almost 66 times lower than the requirement for medical diagnostics (∼5.5 μGy s^–1^), thereby reducing the risk of exposure to high doses of X-rays.^50^ In addition, the dark current drift of the crystal device was measured as 5 × 10^–4^ nA cm^–1^ s^–1^ V^–1^ at 10 V, which would facilitate the potential application of X-ray detection (Figure S12). We also investigated the radiation stability of device **1** under high dose rates and 0 bias voltage, as shown in Figure S13. After a total X-ray dose of 1.2 Gy, the photocurrent remained unchanged, indicating the high operational stability of device **1**. The thermogravimetric result also confirms that **1** exhibits a high thermal stability up to ∼540 K (Figure S14).

## Conclusion

In summary, we report the X-ray LPE in a single-phase perovskite ferroelectric (NPA)_2_(EA)_2_Pb_3_Br_10_ (**1**, NPA = neopentylamine, EA = ethylamine), which has a remarkable spontaneous polarization ∼3.7 μC/cm^2^. In the wavelength range of ordinary visible light, its ferroelectricity gives it a significant LPE. Surprisingly, LPE can also be achieved in the X-ray region. It shows excellent self-powered X-ray responses with sensitivity up to 225 μC Gy^–1^ cm^–2^ and a detection limit of 83.4 nGy s^–1^, being almost 66 times lower than the requirement for medical diagnostics (∼5.5 μGy s^–1^). Furthermore, X-ray LPE can produce a sharp current peak of 16 nA/cm^2^, which is much superior to some heterojunction-based self-powered devices. These findings of the X-ray LPE highlight the potential of hybrid perovskite ferroelectrics toward self-powered X-ray detectors.

## Supporting Information Available

Experimental section and figures, synthesis and single-crystal growth, single-crystal X-ray crystallography and powder X-ray diffraction, characterization, electronic structure calculations, electronic structure calculations, and X-ray detection (PDF)
Crystallographic information on **1** at 200 K (CIF)
Crystallographic information on **1** at 335 K (CIF)
Crystallographic information on **1** at 340 K (CIF)
Transparent Peer Review report available (PDF)

## Supplementary Material

oc3c01274_si_001.pdf

oc3c01274_si_002.cif

oc3c01274_si_003.cif

oc3c01274_si_004.cif
