# Supplementary material for: X-ray-Induced Pyroelectric Effect in a Perovskite Ferroelectric Drives Low Detection Limit Self-Powered Responses
Source: ACS Cent Sci. 2023 Dec 11;9(12):2350–7. doi: 10.1021/acscentsci.3c01274 (PMC10755846; doi:10.1021/acscentsci.3c01274)
Supplement: Supplementary file 1 — oc3c01274_si_001.pdf [file oc3c01274_si_001.pdf]

Supporting Information

X-ray Induced Pyroelectric Effect in a Single-Phase Perovskite Ferroelectric Drives the Low Detection Limit Self-Powered Responses

Yu Ma,[†, §] Wenjing Li,[†, §] Yi Liu,[†] Wuqian Guo,[†, §] Haojie Xu,[†, §] Shiguo Han,[†] Liwei Tang,[†] Qingshun Fan,[†, §] Junhua Luo,[*,†, §] and Zhihua Sun[*,†, §]

[†] State Key Laboratory of Structural Chemistry, Fujian Institute of Research on the Structure of Matter, Chinese Academy of Sciences, Fuzhou, Fujian 350002, People's Republic of China

[§] University of Chinese Academy of Sciences, Chinese Academy of Sciences, Beijing, 100039, People's Republic of China

Experimental Section

Synthesis and single-crystal growth. All the chemical reagents and solvents were purchased and used without further purification. During the synthesis process, the stoichiometric ratio of neopentylamine (10 mmol, 0.87 g), ethylamine (10 mmol, 0.45 g) and $PbBr_2$ (15 mmol, 5.50 g) were slowly added in the solution of concentrated hydrobromic acid. A transparent solution was obtained after the continuous stirring for 60 min at 373 K. Plate-like yellow crystals **1** were obtained by the temperature cooling method after about two weeks, as shown in Figure S1.

Single-Crystal X-ray Crystallography and Powder X-ray Diffraction. X-ray diffraction experiments was carried out for **1** using a Bruker D8 Quesr/Venture diffractometer with the Mo *Kα* radiation (λ = 0.77 Å). The structures were solved by direct methods and confirmed by the full-matrix least-squares refinements on F^2 using the *SHELXTL* software packing. All non-H atoms were refined anisotropically, and all H atoms were generated by geometrical method and refined by using a "riding" model with *Uiso* = 1.2 *Ueq* (C). The above-mentioned structure solution and refinement were conducted in the *Olex*2 software. Crystal data for **1** at 200, 335 and 340 K are listed in Table S1. Deposited CCDC numbers: 2290139-2290141.

Characterization. DSC analysis was carried out using a NETZSCH DSC 200 F3 DSC instrument in the temperature range of 298-420 K. The powder sample

placed in aluminum crucibles was heated and cooled with a rate of 10 K·min^{-1} under the N_2 atmosphere. Single crystal of **1** with the surface deposited by silver paste was used for dielectric constant measurements. The dielectric analysis was performed on TongHui TH2828 analyzer in the temperature range of 298-410 K. The temperature-dependent *P-E* hysteresis loops and *I-E* curves were measured on a ferroelectric analyzer (Radiant Precision Premier II) using the double-wave method. In order to avoid electric discharge at high electric field, single crystal of **1** was immersed in silicone oil to measure the *P-E* hysteresis loops. The UV absorption in solid state was measured at room temperature on a PE Lambda 900 UV-Visible spectrophotometer.

Electronic structure calculations: Electronic structure calculations of **1** were performed by the DFT method by the total-energy code CASTEP, based on the single-crystal structural data. The exchange and correlation effects were treated by Perdew-Burke-Ernzerhof in the generalized gradient approximation. The core-electrons interactions between the ionic cores and the electrons were described by the norm-conserving pseudopotential. The numbers of plane waves included in the basis sets were determined by an energy cutoff 765 eV, and the integration of the Brillouin zone was performed using a Monkhorst-Pack κ-point sampling of 6×6×3.

Photoelectric measurements: Ag electrodes were sputtered on the flat side of a well-polished single crystal. The electrode materials were proven not to have any obvious influence on the photoelectric properties. The photopyroelectric measurements were carried out along the polar *c*- axis of the crystal. The current vs voltage (*I-V*) and photocurrent vs time (*I-t*) with light on or off (measured at zero bias) were measured using a high precision electrometer (Keithley6517B). THORLABS 405, 520, 637, 785, 820 and 980 nm pigtailed laser diode were used for visible light illumination. The incident light intensity was measured by light power meter. The temperature during measurements was controlled at 290 K using a Linkam TS1500 heating stage.

X-Ray Detection. The current-voltage (*I-V*) traces and current-time (*I-t*) curves of **1** were recorded by a Keithley 6517B high-precision electrometer. An Amptek Mini-X2 X-ray tube with silver target (maximum power 4 W) was used

as the X-ray source. The maximum X-ray photons energy is 50 keV and the peak intensity is at 22 keV. The dose rate of X-ray tube was modulated by changing its tube current and measured by a Radcal Accu-Gold X-ray dosimeter attached with the 10×6-180 ion chamber in an integrating mode. We calculated the absorption coefficient and attenuation efficiency of **1** to X-ray radiation using photon cross-section database. The $\mu\tau$ can be calculated using the modified Hecht equation: $I = \frac{I_0 \mu\tau V}{d^2}[1 - \exp\left(-\frac{d^2}{\mu\tau V}\right)]$, where I is the photocurrent, I_0 is the saturated photocurrent, d is the distance between electrodes, and V is the applied bias voltage. The International Union of Pure and Applied Chemistry (IUPAC) defines the dose rate corresponding to an SNR of 3 as the detection limit.

Figures

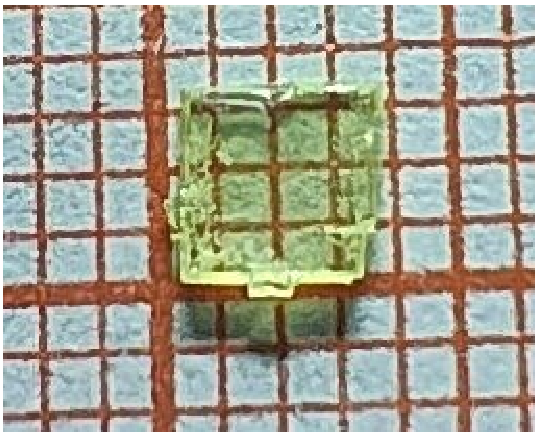

Figure S1. Single crystals of **1** obtained by the temperature-cooling method.

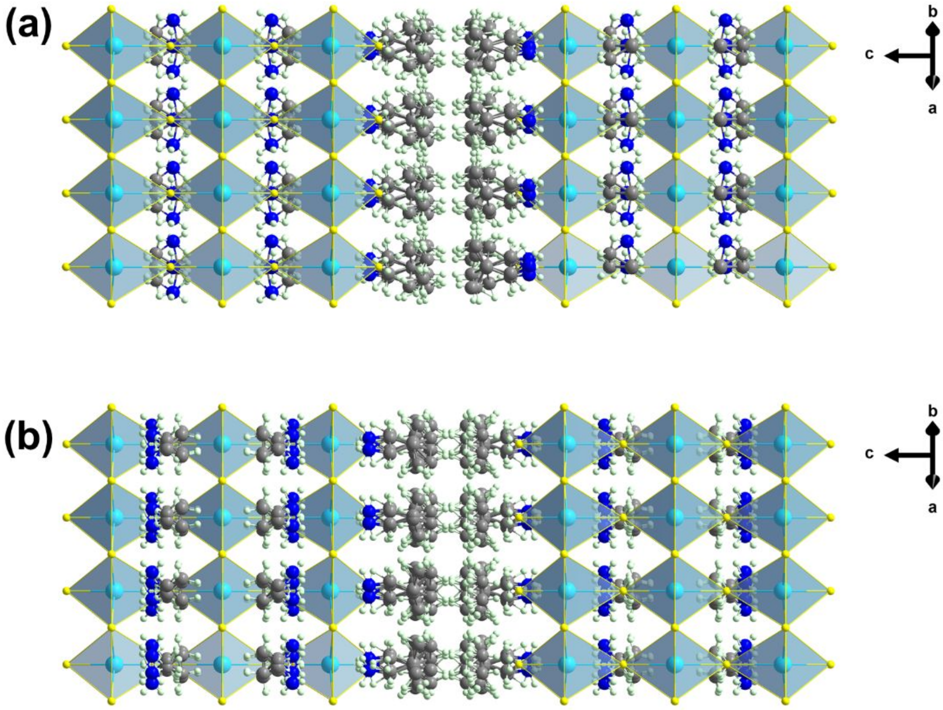

Figure S2. Crystal packing diagram of **1** at 335 K (a) and 340 K (b).

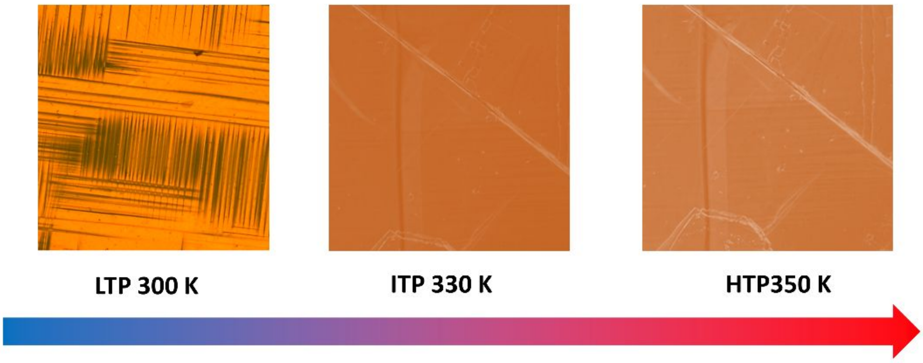

Figure S3. Polarization microscopy images of **1** observed at 300, 330 and 350 K (Slight cracks appear in the crystal during heating process).

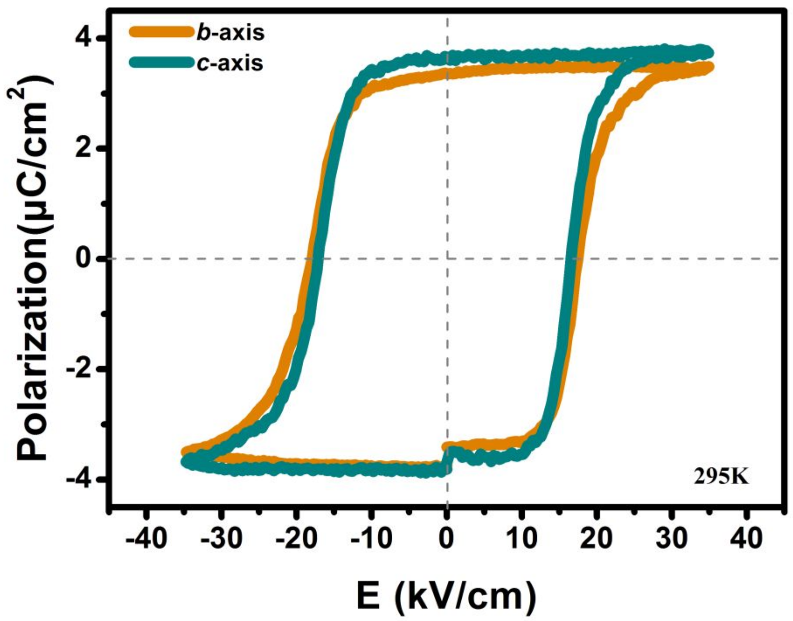

Figure S4. The P-E hysteresis loops measurment of **1** in two different directions. The results prove that **1** is a biaxial ferroelectric.

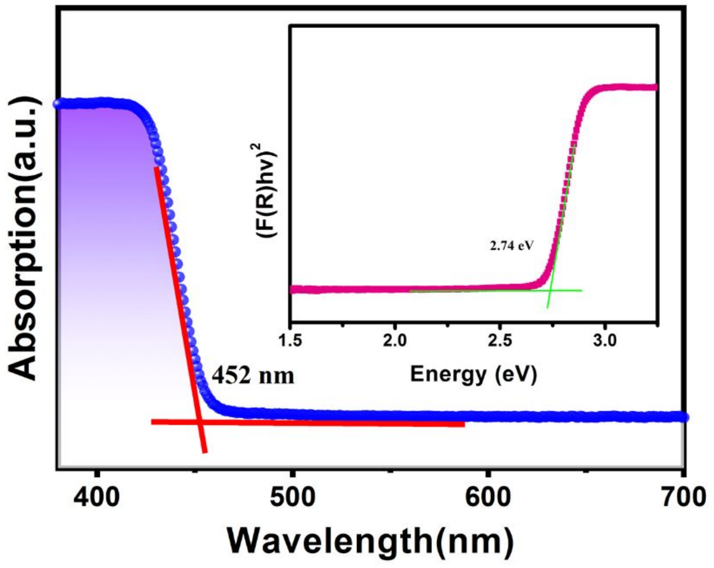

Figure S5. The UV-Vis absorption spectrum of **1**.

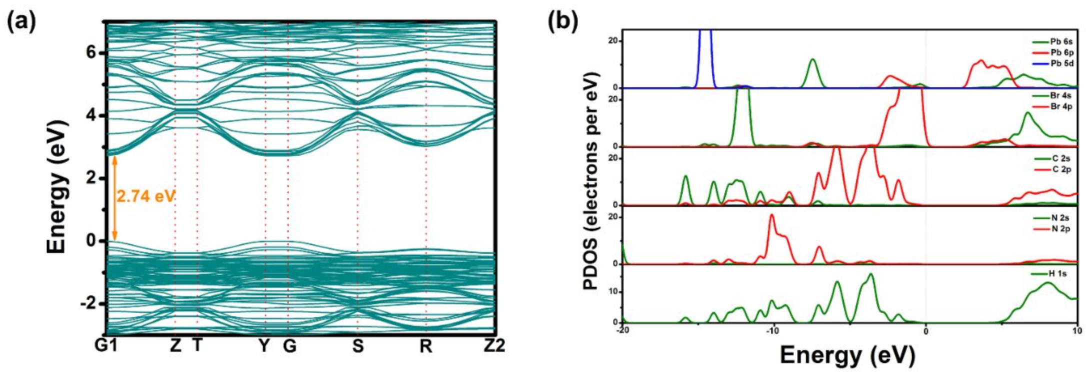

Figure S6. Calculated band structure and PDOS of **1**.

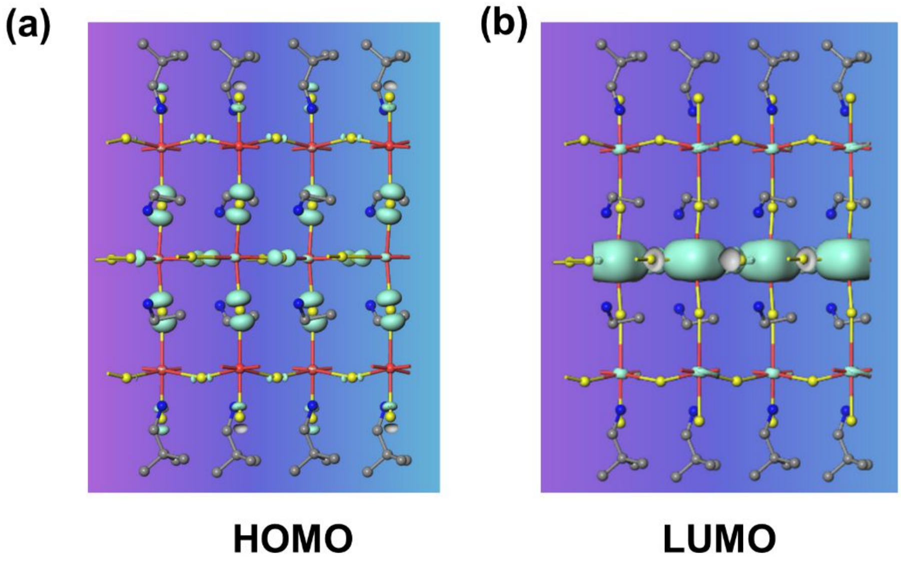

Figure S7. The charge density distribution of **1**.

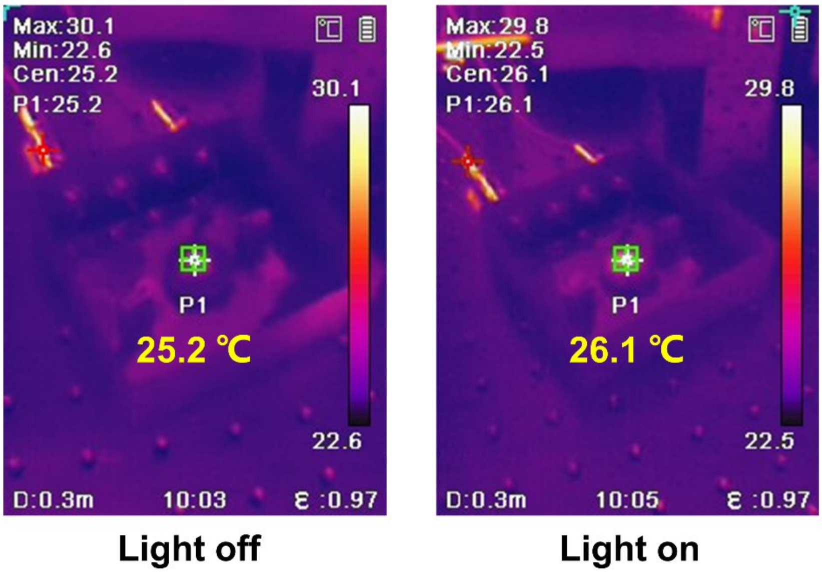

Figure S8. The measurable temperature change in the sample using thermographic techniques.

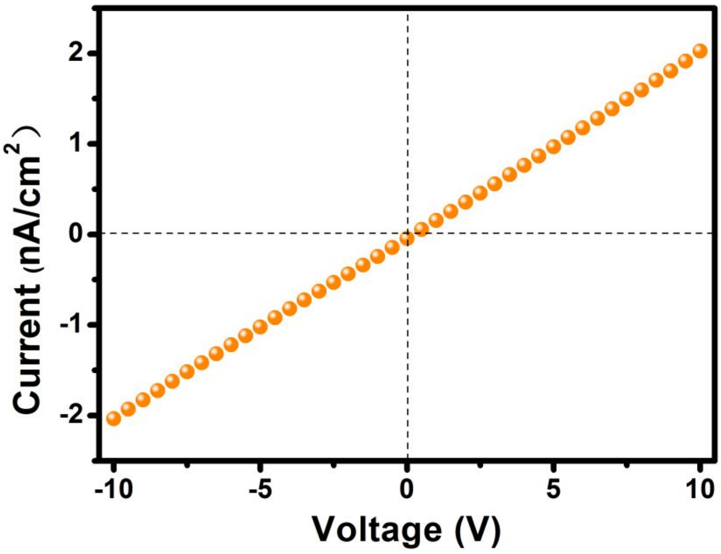

Figure S9. The current density-voltage curve along the single crystal *c*-axis of **1**.

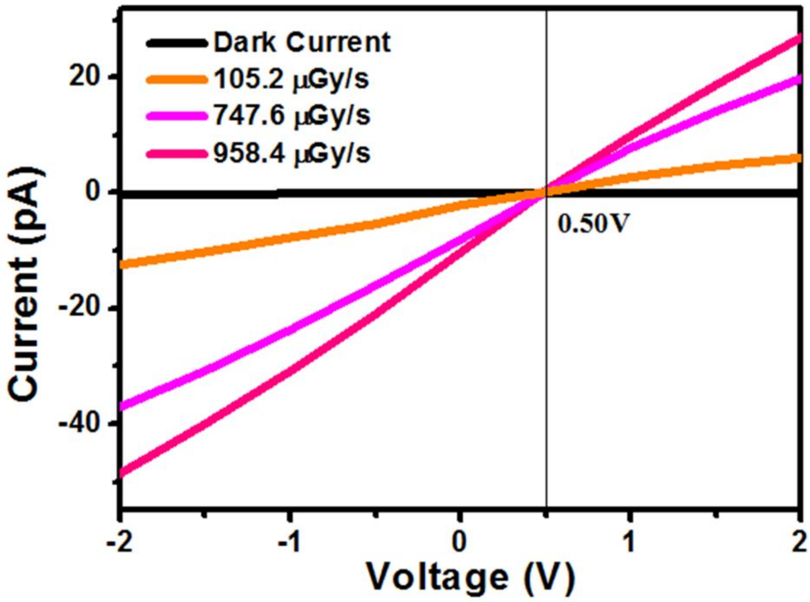

Figure S10. The *I*-*V* curves along the single crystal *c*-axis of **1** under X-ray irradiation.

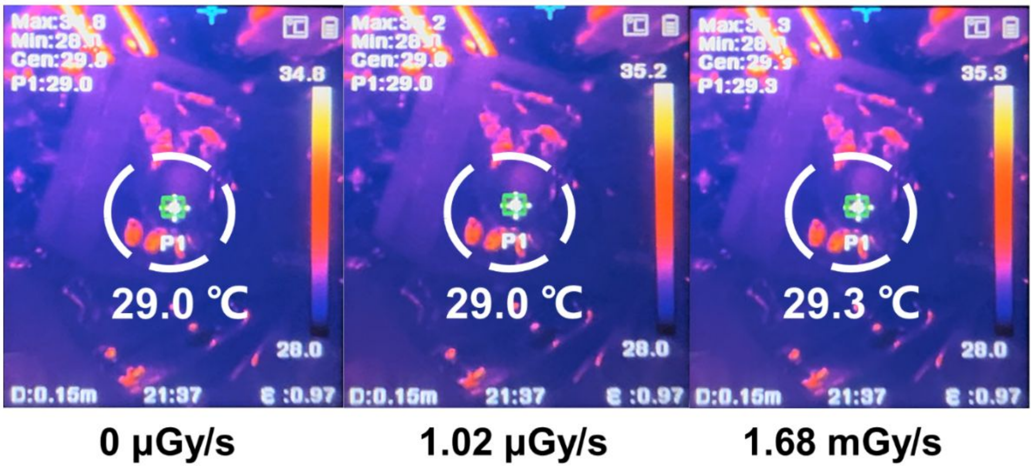

Figure S11. The measurable temperature change in the sample using thermographic techniques under X-ray irradiation.

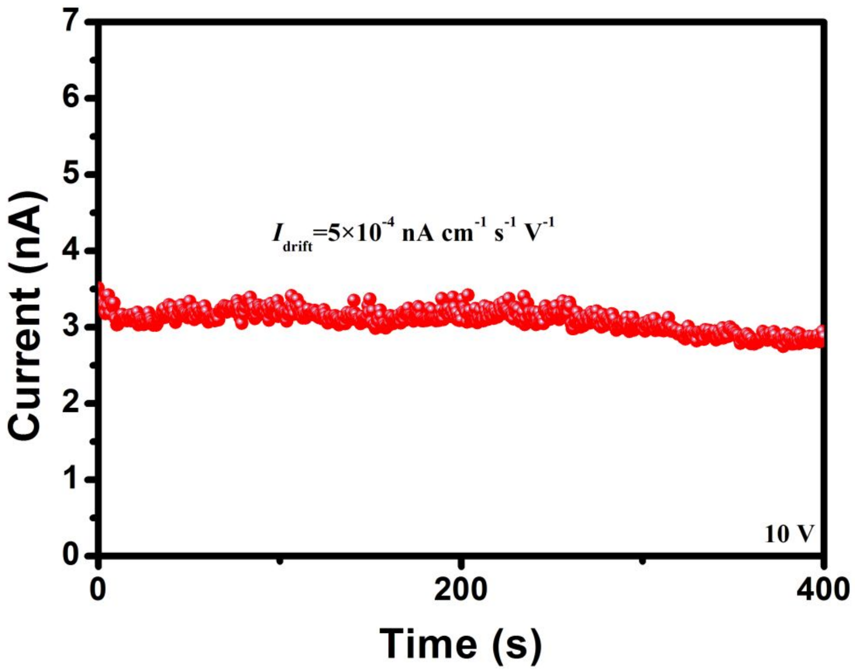

Figure S12. The Dark current tracking of detector at 10 V bias.

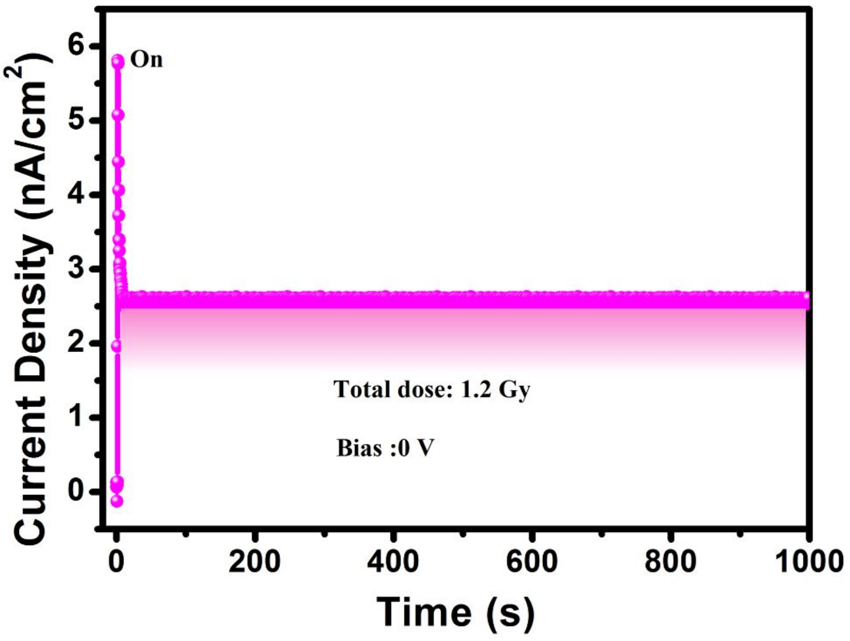

Figure S13. The high operational stability of device **1.**

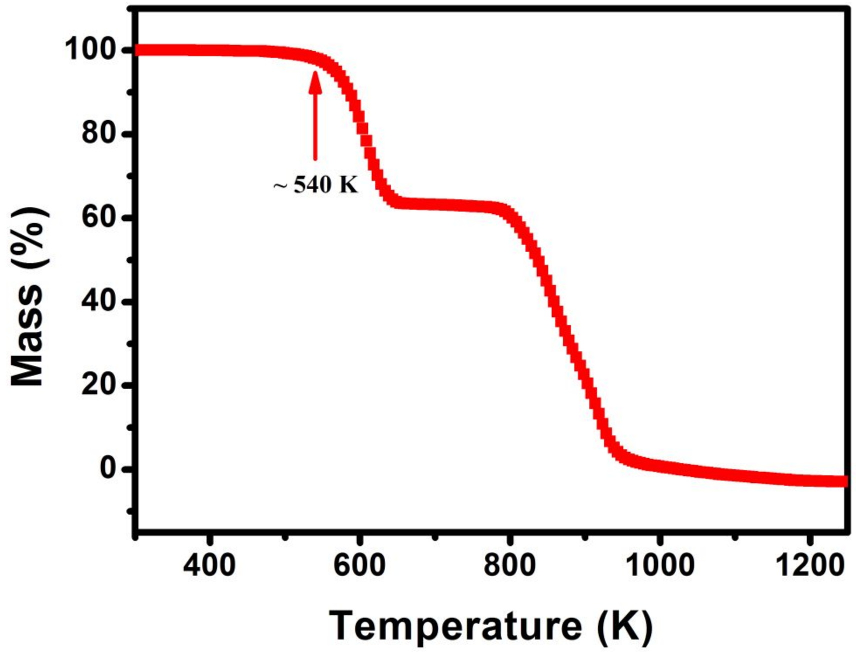

Figure S14. Thermogravimetric analysis curve of **1**.

Table S1. Crystal data for **1** collected at 200, 335 and 340 K, respectively.

Empirical formula	$C_{14}H_{44}Br_{10}N_4Pb_3$	$C_{14}H_{44}Br_{10}N_4Pb_3$	$C_{14}H_{44}Br_{10}N_4Pb_3$
Formula weight	1689.20	1689.20	1689.20
Temperature/K	200	335	340
Crystal system	orthorhombic	tetragonal	tetragonal
Space group	$Cmc2_1$	$I4/m$	$I4/mmm$
a/Å	52.054(4)	6.0348(4)	6.0394(2)
b/Å	8.4472(7)	6.0348(4)	6.0394(2)
c/Å	8.4691(7)	52.485(4)	52.515(2)
Volume/Å^3	3724.0(5)	1911.4(3)	1915.47(15)
Z	4	2	2
ρ_{calc} (g/cm^3)	3.013	2.935	2.929
μ/mm^{-1}	24.272	23.664	23.594
F(000)	3008.0	1504.0	1504.0
2Θ range for data collection/°	4.696 to 49.986	6.21 to 54.95	4.654 to 55.088
Index ranges	$-61 \le h \le 61$, $-10 \le k \le 10$, $-10 \le l \le 10$	$-6 \le h \le 7$, $-7 \le k \le 7$, $-68 \le l \le 68$	$-7 \le h \le 6$, $-7 \le k \le 7$, $-67 \le l \le 67$
Reflections collected	21210	9292	6210
Independent reflections	3297 [R_{int} = 0.0964, R_{sigma} = 0.0664]	1127 [R_{int} =0.0661, R_{sigma} = 0.0418]	745 [R_{int} = 0.0617, R_{sigma} = 0.0366]
Data/restraints/parameters	3297/107/151	1127/112/108	745/22/61
Goodness-of-fit on F^2	1.036	1.050	1.064
Final R indexes [I>=2σ (I)]	R_1 = 0.0641, wR_2 = 0.1934	R_1 = 0.0491, wR_2 = 0.1268	R_1 = 0.0438, wR_2 = 0.1151
Final R indexes [all data]	R_1 = 0.0801, wR_2 = 0.2063	R_1 = 0.0705, wR_2 = 0.1415	R_1 = 0.0566, wR_2 = 0.1240

Table S2. Selected Pb-Br bond lengths of crystal **1** at 200 K.

Atom	Atom	Length/Å	Atom	Atom	Length/Å
Pb2	Br3	3.283(4)	Pb1	Br3[5]	2.993(3)
Pb2	Br5	3.015(4)	Pb1	Br3	2.993(3)
Pb2	Br5[1]	3.019(4)	C3	N2	1.46(3)
Pb2	Br4[2]	3.009(5)	C3	C4	1.47(3)
Pb2	Br4	3.006(5)	C7	C4	1.63(7)
Pb2	Br6	2.776(4)	C6	C4	1.49(6)
Pb1	Br2	3.036(5)	C5	C4	1.50(3)
Pb1	Br2[3]	3.173(5)	N1	C1	1.50(3)
Pb1	Br1[4]	3.101(4)	C1	C2	1.50(3)
Pb1	Br1	2.949(6)			

[1]+X,2-Y,-1/2+Z; [2]+X,1-Y,-1/2+Z; [3]1-X,1-Y,1/2+Z; [4]1-X,2-Y,1/2+Z; [5]1-X,+Y,+Z

Table S3 Bond Angles for **1** at 200K.

Atom	Atom	Atom	Angle/°	Atom	Atom	Atom	Angle/°
Br5	Pb2	Br3	101.47(13)	Br1	Pb1	Br3	88.99(13)
Br5[1]	Pb2	Br3	100.90(14)	Br3[5]	Pb1	Br2	83.73(9)
Br5	Pb2	Br5[1]	89.17(3)	Br3[5]	Pb1	Br2[3]	90.24(12)
Br4[2]	Pb2	Br3	83.85(13)	Br3	Pb1	Br2	83.73(9)
Br4	Pb2	Br3	84.28(14)	Br3	Pb1	Br2[3]	90.24(12)
Br4[2]	Pb2	Br5	174.57(12)	Br3	Pb1	Br1[4]	96.26(9)
Br4[2]	Pb2	Br5[1]	88.79(16)	Br3[5]	Pb1	Br1[4]	96.26(9)
Br4	Pb2	Br5	92.05(15)	Br3[5]	Pb1	Br3	167.4(2)
Br4	Pb2	Br5[1]	174.34(13)	Pb1	Br2	Pb1[6]	159.2(2)
Br4	Pb2	Br4[2]	89.50(3)	Pb1	Br1	Pb1[7]	151.0(2)
Br6	Pb2	Br3	166.72(13)	Pb1	Br3	Pb2	170.80(16)
Br6	Pb2	Br5[1]	88.29(19)	Pb2	Br5	Pb2[8]	161.97(17)

Br6	Pb2	Br5	88.11(19)	Pb2	Br4	Pb2[9]	172.68(18)
Br6	Pb2	Br4[2]	86.8(2)	N2	C3	C4	138(5)
Br6	Pb2	Br4	86.23(19)	C3	C4	C7	112(4)
Br2	Pb1	Br2[3]	87.34(5)	C3	C4	C6	117(4)
Br2	Pb1	Br1[4]	178.90(19)	C3	C4	C5	108(4)
Br1	Pb1	Br2[3]	172.91(15)	C6	C4	C7	104(4)
Br1[4]	Pb1	Br2[3]	93.76(16)	C6	C4	C5	112(4)
Br1	Pb1	Br2	85.57(16)	C5	C4	C7	103(4)
Br1	Pb1	Br1[4]	93.34(6)	C2	C1	N1	99(4)
Br1	Pb1	Br3[5]	88.99(13)				

[1]+X,2-Y,-1/2+Z; [2]+X,1-Y,-1/2+Z; [3]1-X,1-Y,1/2+Z; [4]1-X,2-Y,1/2+Z; [5]1-X,+Y,+Z; [6]1-X,1-Y,-1/2+Z; [7]1-X,2-Y,-1/2+Z; [8]+X,2-Y,1/2+Z; [9]+X,1-Y,1/2+Z
