# Supplementary material for: X-ray-Induced Pyroelectric Effect in a Perovskite Ferroelectric Drives Low Detection Limit Self-Powered Responses
Source: ACS Cent Sci. 2023 Dec 11;9(12):2350–7. doi: 10.1021/acscentsci.3c01274 (PMC10755846; doi:10.1021/acscentsci.3c01274)
Supplement: Supplementary file 5 — oc3c01274_si_005.pdf [file oc3c01274_si_005.pdf]

oc-2023-01274d.R1

Name: Peer Review Information for "X-ray Induced Pyroelectric Effect in a Perovskite Ferroelectric Drives Low Detection Limit Self-Powered Responses"

First Round of Reviewer Comments

Reviewer: 1

Comments to the Author

The authors report for the first time the photopyroelectric effect induced by X-ray in single-phase perovskite ferroelectrics, and realize self-driven detection with low detection limits. And X-ray-induced photopyroelectric effect can generate a sharp current peak ~16 nA/cm2, which is nearly two orders of magnitude better than some heterojunction-based self-powered devices. This work not only develops a new mode for X-ray detection, but also provides valuable insights for future photoelectric device application. This work will attract great attention from readers. I recommend publishing this article after minor revision.

1. In manuscript, the structural analysis follows the Aizu symmetry breaking of 4/mmmFmm2 during the phase transition. That is, this is a biaxial ferroelectric, can the author provide a hysteresis loop measurement result in the other direction.

2. For the measurement of crystal photopyroelectric, the polarization direction of the crystal is crucial, and the authors need to provide the test direction of the crystal.

3. The author provides cross-stripe domain patterns at 300 K and 350 K to demonstrate phase transitions, and intermediate temperature phase cross-stripe domain pattern should also be provided.

4. For self-powered X-ray detection, dark current drift is an important parameter to measure its stability. The author should provide it in the manuscript.

Reviewer: 2

Comments to the Author

The authors reported X-ray light-induced pyroelectric effect in a single-phase ferroelectric of (NPA)2(EA)2Pb3Br10, adopting a two-dimensional trilayered perovskite motif. The reported compound has a large spontaneous polarization of ~3.7 μC/cm2. The authors used several measurements such as single crystal X-ray diffraction analysis, DSC, dielectric and SHG measurements, and so on to investigate its physical properties. The article has detailed data and clear logic. Thus, I suggest the publication of this article in ACS Central Science after minor revision.

1. The resolution of Fig. S3 should be improved.

2. How about the thermal stability of this compound?

3. A two-dimensional multiaxial lead-free ferroelectric should also be discussed in this article (National Science Review, Volume 8, Issue 5, 2021, nwaa232, https://doi.org/10.1093/nsr/nwaa232).

Author's Response to Peer Review Comments:

Dear Prof. Editor,

Thanks for your and the reviewers' appraisements and comments on our manuscript (Manuscript ID: **oc-2023-01274d**). We are very grateful to their suggestion on how to make the statements well for our paper to be suitable for publication in ***ACS Cent. Sci.*** Our manuscript has been revised carefully according to the reviewers' suggestion, and the point-by-point responses to the comments are attached in this letter. Moreover, all the changes have been highlighted by giving the text with a yellow background in the revision. We believe that we have adequately addressed the reviewers' concerns, and hope that the revision would satisfy the publishable standard of your esteemed journal.

With my best wishes！

Sincerely, Yours

Zhihua Sun

Professor of Chemistry

Fujian Institute of Research on the Structure of Matter,

Chinese Academy of Sciences, Fuzhou, Fujian 350002, China

E-mail: sunzhihua@fjirsm.ac.cn

Response to Reviewers

Reviewer: 1

The authors report for the first time the photopyroelectric effect induced by X-ray in single-phase perovskite ferroelectrics, and realize self-driven detection with low detection limits. And X-ray-induced photopyroelectric effect can generate a sharp current peak ~16 nA/cm^2, which is nearly two orders of magnitude better than some heterojunction-based self-powered devices. This work not only develops a new mode for X-ray detection, but also provides valuable insights for future photoelectric device application. This work will attract great attention from readers. I recommend publishing this article after minor revision.

Q1. In manuscript, the structural analysis follows the Aizu symmetry breaking of 4/*mmm*F*mm*2 during the phase transition. That is, this is a biaxial ferroelectric, can the author provide a hysteresis loop measurement result in the other direction.

√ Answer: Thanks for the reviewer's useful suggestion. We have provided the hysteresis loop measurement results along two different crystalline directions in the support information (*b*- and *c*- axes).

Supporting Information, Figure S4

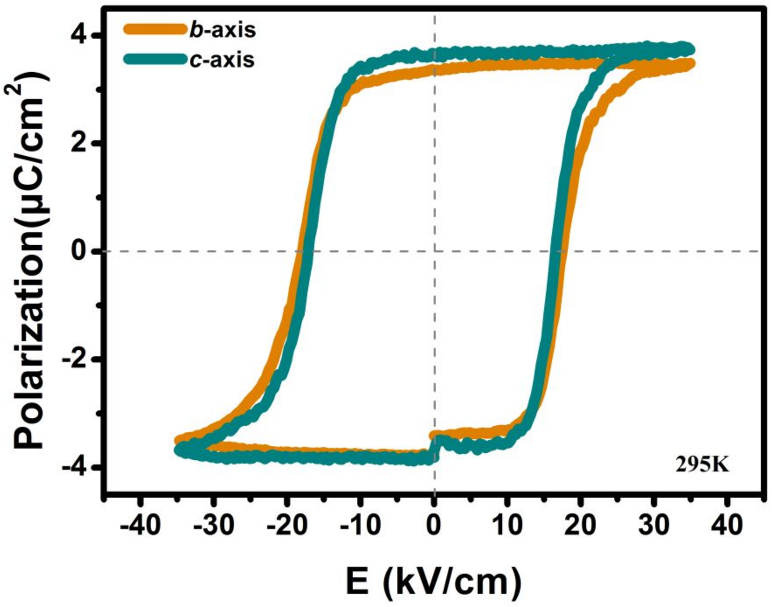

Figure S4. *The P-E hysteresis loops measurment of **1** in two different directions. The results prove that **1** is a biaxial ferroelectric.*

Q2. For the measurement of crystal photopyroelectric, the polarization direction of the crystal is crucial, and the authors need to provide the test direction of the crystal.

√Answer: Thanks for the reviewer's useful suggestion. We have added the direction of the crystal photopyroelectric test in the support information.

Supporting Information, **Photoelectric measurements**

The photopyroelectric measurements were carried out along the polar c- axis of the crystal.

Q3. The author provides cross-stripe domain patterns at 300 K and 350 K to demonstrate phase transitions, and intermediate temperature phase cross-stripe domain pattern should also be provided.

√Answer: Thanks for the reviewer's useful suggestion. We have provided the higher-quality cross-stripe domain patterns at different temperatures in Figure S3. And the intermediate temperature phase cross-stripe domain pattern was also provided. The cross-stripe domain patterns that vanish at temperatures over 305 K, which is consistent with the ferroelectric phase transition.

Page 4, Paragraph 1

Furthermore, using polarizing microscopy to observe cross-stripe domain patterns that vanish at temperatures over 305 K, which is consistent with the ferroelectric phase transition (Figure S3).

Supporting Information, Figure S3

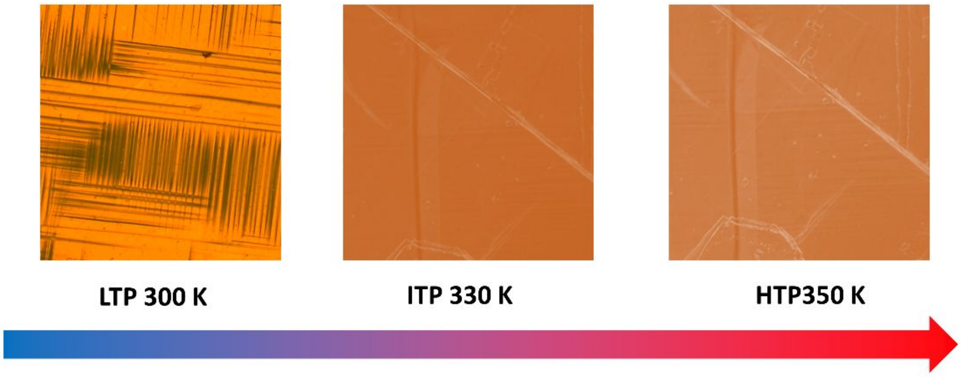

Figure S3. *Polarization microscopy images of **1** observed at 300, 330 and 350 K (Slight cracks appear in the crystal during heating process).*

Q4. For self-powered X-ray detection, dark current drift is an important parameter to measure its stability. The author should provide it in the manuscript.

√ **Answer:** Thanks for the reviewer's useful suggestion. We have provided the dark current drift in the manuscript.

Supporting Information, Figure S12

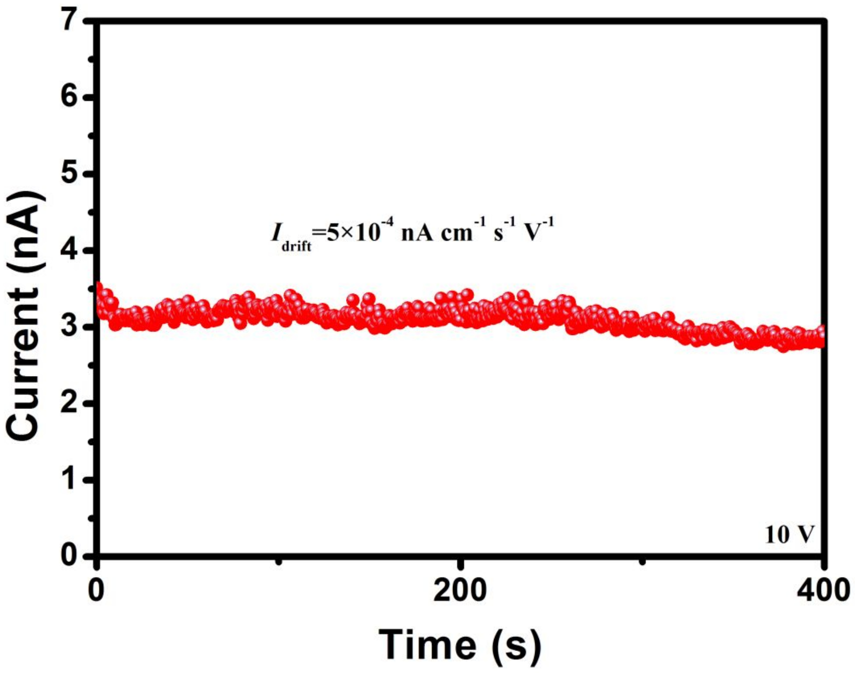

Figure S12. *The Dark current tracking of detector at 10 V bias.*

Page 6, Paragraph 2

In addition, the dark current drift of the crystal device was measured as 5 × 10^{-4} nA cm^{-1} s^{-1} V^{-1} at 10 V, which would facilitate the potential application of X-ray detection.

Reviewer: 2

The authors reported X-ray light-induced pyroelectric effect in a single-phase ferroelectric of $(NPA)_2(EA)_2Pb_3Br_{10}$, adopting a two-dimensional trilayered perovskite motif. The reported compound has a large spontaneous polarization of ~3.7 μC/cm2. The authors used several measurements such as single crystal X-ray diffraction analysis, DSC, dielectric and SHG measurements, and so on to investigate its physical properties. The article has detailed data and clear logic. Thus, I suggest the publication of this article in ACS Central Science after minor revision.

Q1. The resolution of Fig. S3 should be improved.

√Answer: Thanks for the reviewer's useful suggestion. We have provided the higher-quality cross-stripe domain patterns at different temperatures in Figure S3. And intermediate temperature phase cross-stripe domain pattern was also provided. The cross-stripe domain patterns that vanish at temperatures over 305 K, which is consistent with the ferroelectric phase transition.

Page 4, Paragraph 1

Furthermore, using polarizing microscopy to observe cross-stripe domain patterns that vanish at temperatures over 305 K, which is consistent with the ferroelectric phase transition (Figure S3).

Supporting Information, Figure S3

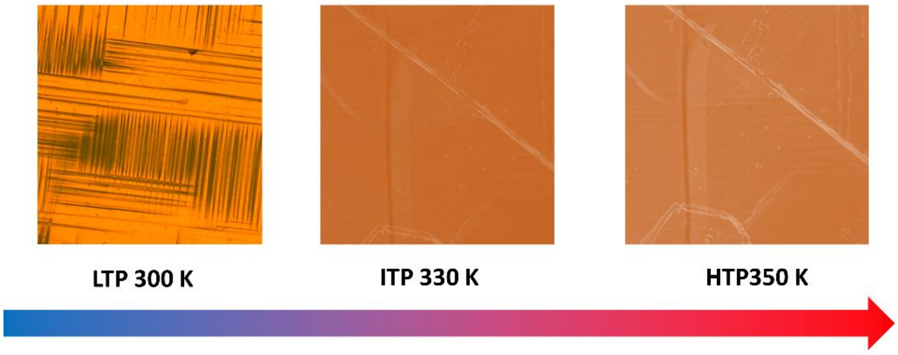

Figure S3. *Polarization microscopy images of* ***1*** *observed at 300, 330 and 350 K (Slight cracks appear in the crystal during heating process).*

Q2. How about the thermal stability of this compound?

√Answer: Thanks for the reviewer's useful suggestion. The thermogravimetric result confirms that compound exhibits a high thermal stability up to ~540 K.

Supporting Information, Figure S14

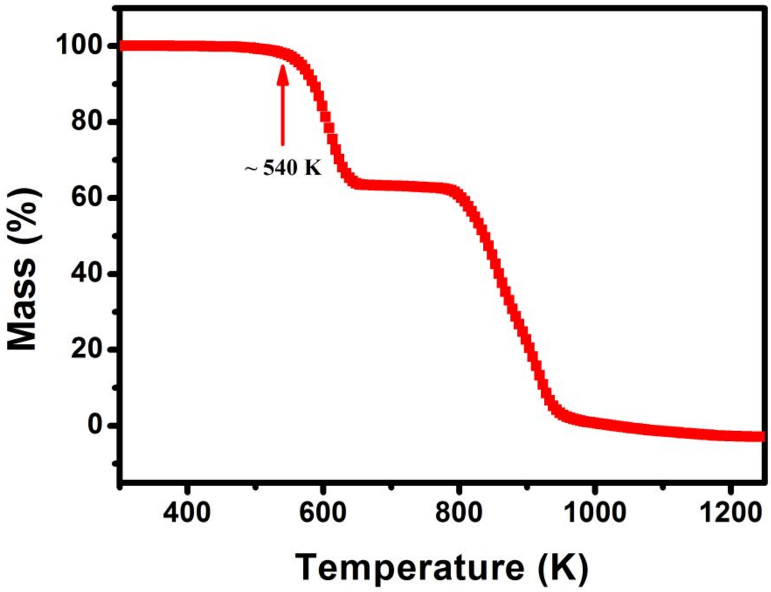

Figure S14. *Thermogravimetric analysis curve of* ***1****.*

Page 6, Paragraph 2

*And the thermogravimetric result also confirms that **1** exhibits a high thermal stability up to ~540 K (Figure S14).*

Q3. A two-dimensional multiaxial lead-free ferroelectric should also be discussed in this article (National Science Review, Volume 8, Issue 5, 2021, nwaa232, https://doi.org/10.1093/nsr/nwaa232).

√ **Answer:** Thanks for the reviewer's useful suggestion. We have discussed two-dimensional multiaxial lead-free ferroelectric in this article.

Page 3, Paragraph 2

These distortions break the inversion symmetry and promote the formation of molecular dipoles, as well as the electric polarization, similar to the lead-free ferroelectric [3,3-difluorocyclobutylammonium]$_2$CuCl$_4$ (0.29 μC/cm^2).[30-32]

REFERENCES

32. Huang, C.-R.; Luo, X.; Chen, X.-G.; Song, X.-J.; Zhang, Z.-X.; Xiong, R.-G., A multiaxial lead-free two-dimensional organic-inorganic perovskite ferroelectric. Natl. Sci. Rev. ***2021,*** *8, nwaa232.*
